# Supplementary material for: Exploring the metabolic potential of Aeromonas to utilise the carbohydrate polymer chitin
Source: RSC Chem Biol. 2024 Dec 9;6(2):227–39. doi: 10.1039/d4cb00200h (PMC11653859; doi:10.1039/d4cb00200h)
Supplement: CB-006-D4CB00200H-s005 [file CB-006-D4CB00200H-s005.pdf]

Supplementary Information (SI) for RSC Chemical Biology.
This journal is © The Royal Society of Chemistry 2024

Supplementary information material to:
Exploring the metabolic potential of *Aeromonas* to utilise the carbohydrate polymer chitin

Claudia G. Tugui[1], Dimitry Y. Sorokin[1,2], Wim Hijnen[3], Julia Wunderer[3], Kaatje Bout[1], Mark C.M. van Loosdrecht[1] & Martin Pabst[1]*

[1]Delft University of Technology, Department of Biotechnology, Delft, The Netherlands.

[2]Winogradsky Institute of Microbiology, Federal Research Centre of Biotechnology, RAS, Moscow, Russia.

[3]Evides Water Company, Rotterdam, The Netherlands.

*Contact: m.pabst@tudelft.nl

TABLE OF CONTENTS **PAGE**

SI Figure 1: OD660 of *A. bestiarum* and *A. rivuli* cultures grown on glucose and chitin. 2
SI Figure 2: Microscopy images of the *A. bestiarum* and *A. rivuli* grown on glucose and chitin. 3
Si Figure 3: PCA analysis of replicate proteome profiles for *A. bestiarum* and *A. rivuli*. 4
SI Figure 4: Hierarchical clustering of replicate proteome profiles for *A. bestiarum* and *A. rivuli*. 5
SI Figure 5: Chitin degradation assay with A. bestiarum and A. rivuli cell culture supernatants. 6
SI Figure 6: Evaluation of database searching accuracy. 7
SI Table 1: Evaluation of database searching accuracy for *A. bestiarum* (grown on glucose). 7
SI Table 2: Evaluation of database searching accuracy for *A. rivuli* (grown on glucose). 7
SI Figure 7: CAZy and binding-proteins involved in degradation of chitin for different Aeromonas strains. 8
SI Table 3: CAZy families potentially involved in the degradation of different biopolymers. 9

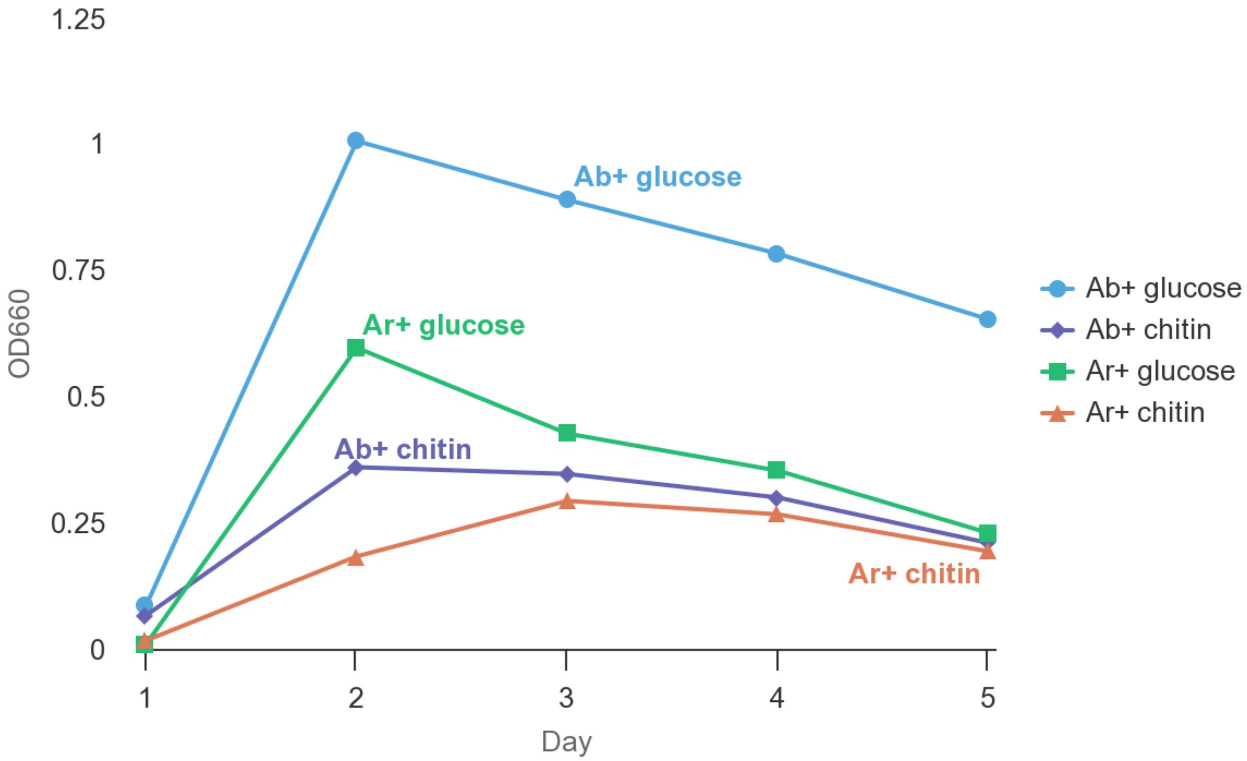

SI Figure 1. OD660 measurements for *A. bestiarum* (Ab) and *A. rivuli* (Ar) cultures grown on glucose or chitin over 5 days.

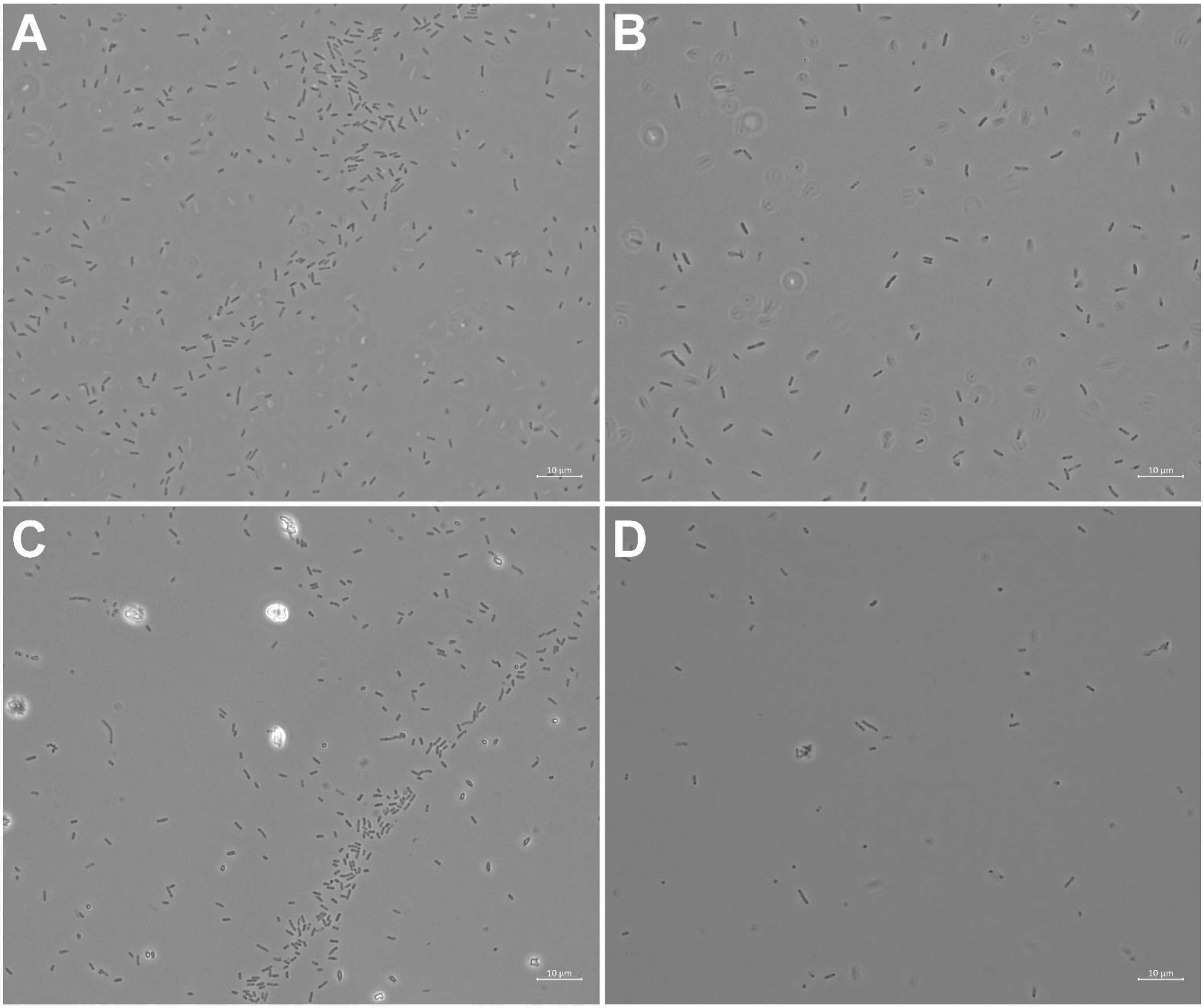

SI Figure 2. Light microscopy images (at 100x magnification) of cultures from (A) *A. bestiarum* grown on chitin, (B) *A. bestiarum* grown on glucose, (C) *A. rivuli* grown on glucose, and (D) *A. rivuli* grown on chitin.

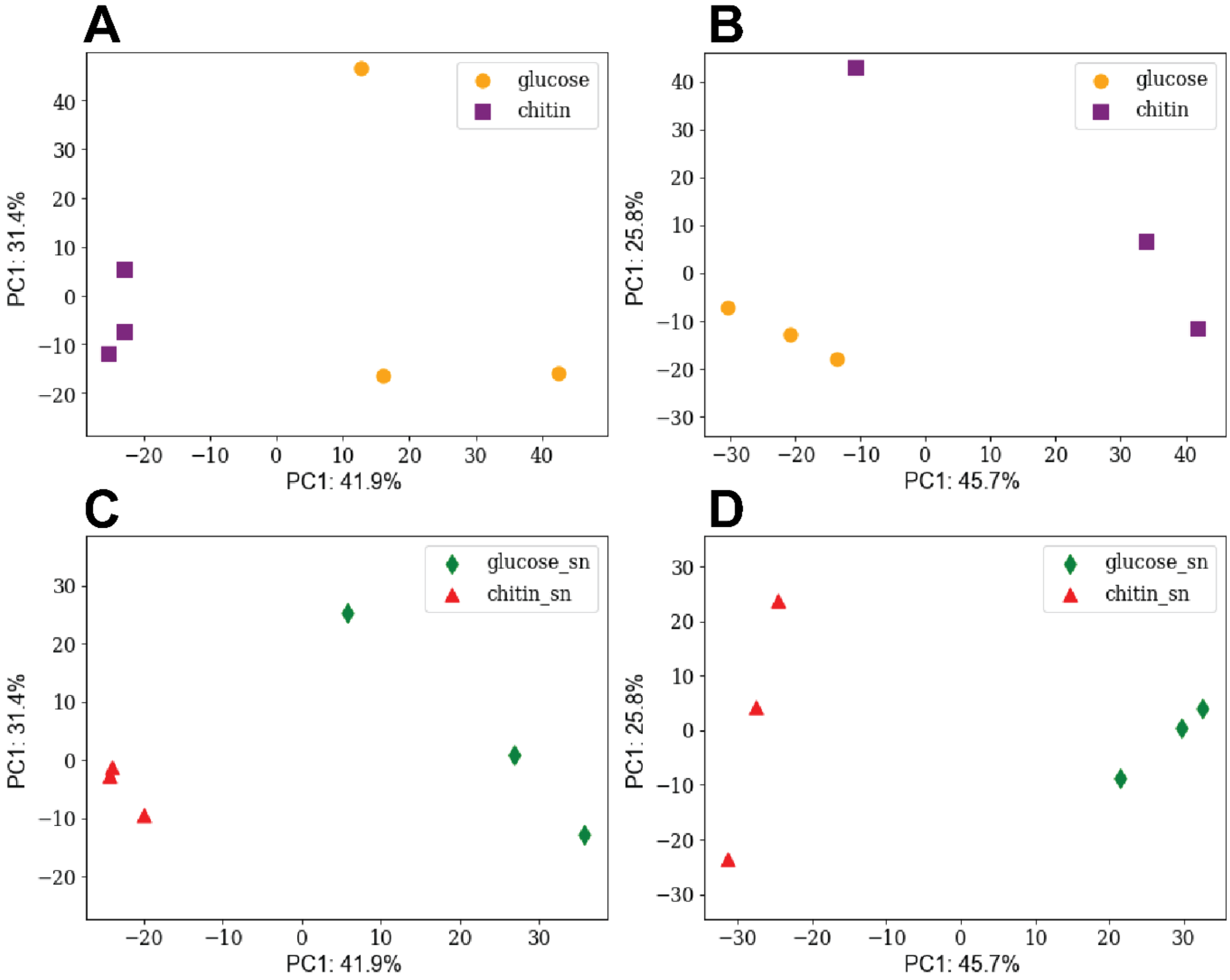

SI Figure 3: The graphs display the principal component analysis (PCA) of the profiles acquired from the triplicate growth experiments of (A) *A. bestiarum* biomass, (B) *A. rivuli* biomass, (C) *A. bestiarum* secretome, and (D) *A. rivuli* secretome. Supernatant is abbreviated as "sn".

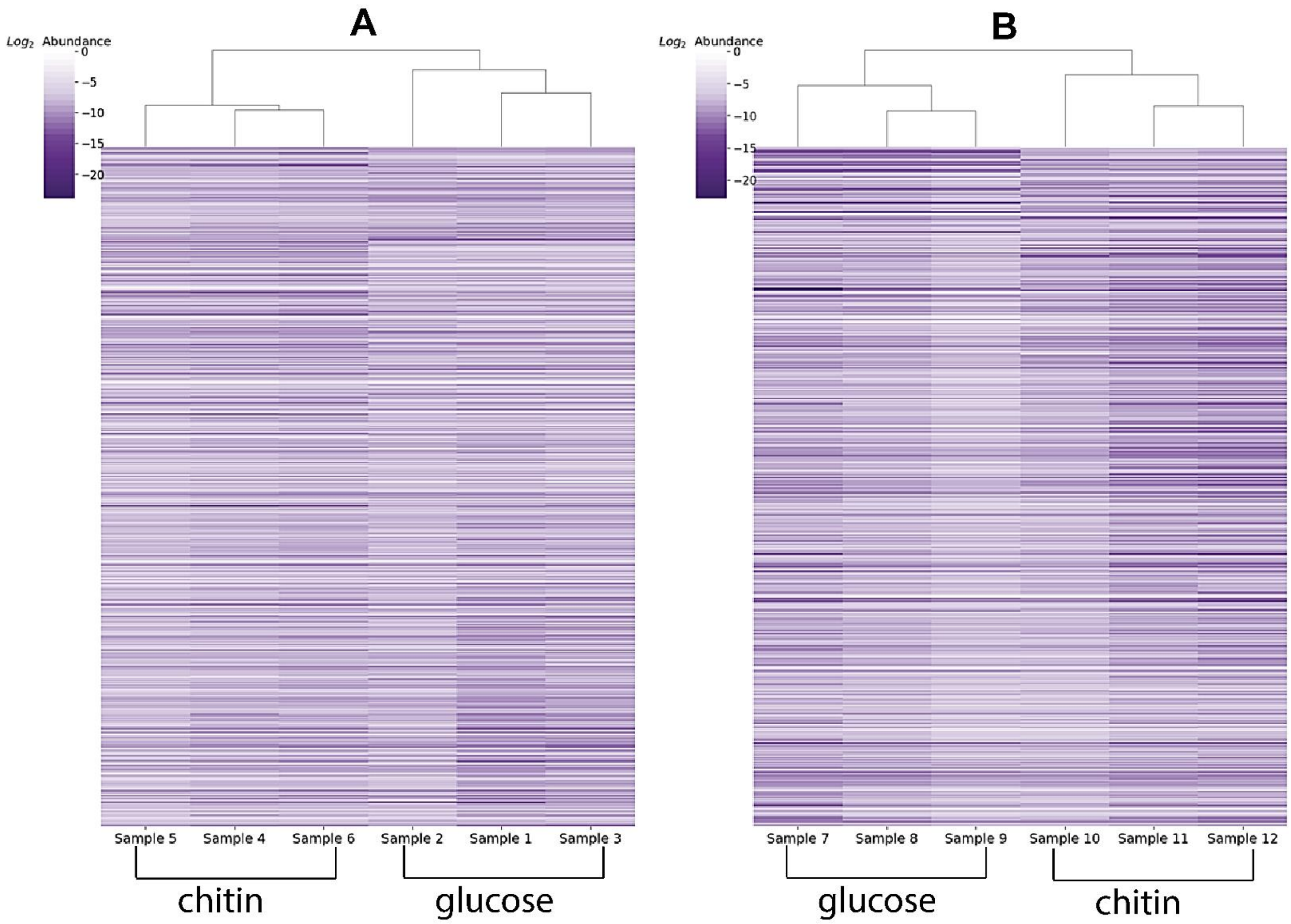

SI Figure 4: The heatmaps display the hierarchical clustering of the cellular proteome profiles acquired from the triplicate growth experiments of (A) *A. bestiarum*, and (B) *A. rivuli*.

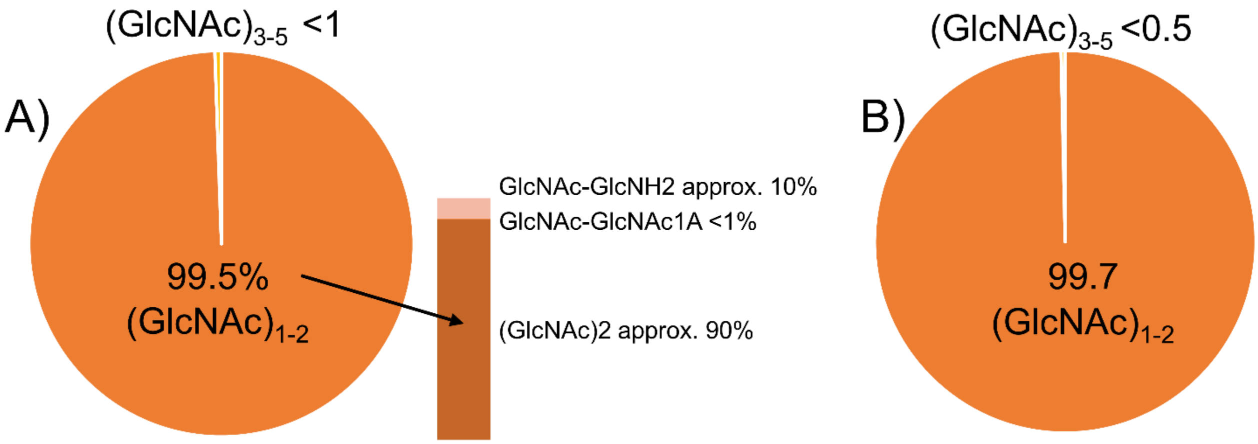

SI Figure 5: The pie charts display the distribution between different GlcNAc hydrolysis products (monomers = $(GlcNAc)_1$, dimers = $(GlcNAc)_2$, trimers = $(GlcNAc)_3$, and tetramers = $(GlcNAc)_4$) obtained by exposing chitin to the cell culture supernatants of A) *A. bestiarum* and B) *A. rivuli*. The abundances are based on the different fragment ion intensities of the respective hydrolysis products. Interestingly, the ratios $(GlcNAc)_2$ to $(GlcNAc)_1$ is inverted between both Aeromonas species. For both strains small quantities of oxidized forms could be detected. However, all were <1% in abundance compared to the main hydrolysis product. For *A. bestiarum* partially deacetylated forms could also be detected (approx. 10%). The m/z values for the native hydrolysis products are: GlcNAc = 222.09721, C8H16NO6+; GlcNAc-GlcNAc = C16H29N2O11+, 425.17659; and for the oxidized forms are: GlcNAc1A = 238.09213, C8H16NO7+; GlcNAc-GlcNAc1A = C16H29N2O12+, 441.1715, and for the native deacetylated forms are: GlcNAc-GlcNH2 = 383.16602, C14H27N2O10+.

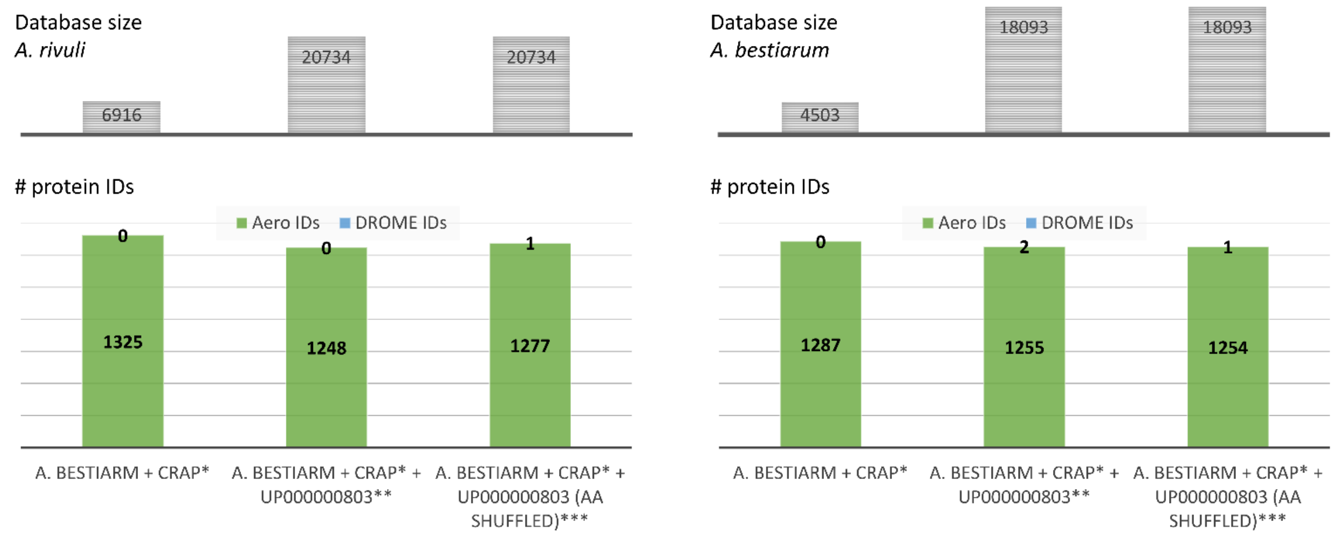

SI Figure 6: Evaluation of database searching accuracy. After increasing the database size from 4,503 to 18,093 entries for *A. bestiarum* and from 6,916 to 20,734 entries for *A. rivuli* (following the incorporation of the reference proteome UP000000803 from Drosophila melanogaster (DROME), either directly or after randomizing the amino acid sequence), the number of identified proteins decreased only slightly, by an average of 2.52% for *A. bestiarum* and 4.75% for *A. rivuli*. Additionally, the number of incorrect matches to the decoy DROME proteome remained below 1% for both strains, averaging 0.12% for A. bestiarum and 0.04% for A. rivuli.

SI Table 1: Evaluation of database searching accuracy for *A. bestiarum* (grown on glucose).

A. bestiarum **(pellet, grown on Glc)**	**$DB size**	**PSMs (total)**	**PSMs (DROME #)**	**#Protein IDs (total)**	**#Protein IDs (DROME)**	**% less Protein IDs**	**PSM FDR (PEAKS)**	**Protein FDR (PEAKS)**	**% DROME IDs**
A. bestiarm **+ cRAP***	4503	30139	0	1287	0	0.00%	1.0	0.4	X
A. bestiarm **+ cRAP* + UP000000803****	18093	28117	5	1255	2	2.49%	1.0	0.0	0.16%
A. bestiarm **+ cRAP* + UP000000803 (AA shuffled)*****	18093	27918	2	1254	1	2.56%	1.0	0.0	0.08%

SI Table 2: Evaluation of database searching accuracy for *A. rivuli* (grown on glucose).

A. rivuli (pellet, **grown on Glucose)**	**$DB size**	**PSMs (total)**	**PSMs (DROME #)**	**#Protein IDs (total)**	**#Protein IDs (DROME)**	**% less Protein IDs**	**PSM FDR (PEAKS)**	**Protein FDR (PEAKS)**	**% DROME IDs**
A. rivuli **+ cRAP***	6916	13638	0	1325	0	0	1	0.1	X
A. rivuli **+ cRAP* + UP000000803****	20734	12888	0	1248	0	5.81%	1.0	0.0	0.00%
A. rivuli **+ cRAP* + UP000000803 (AA shuffled)*****	20734	13327	2	1277	1	3.62%	1.0	0.0	0.08%

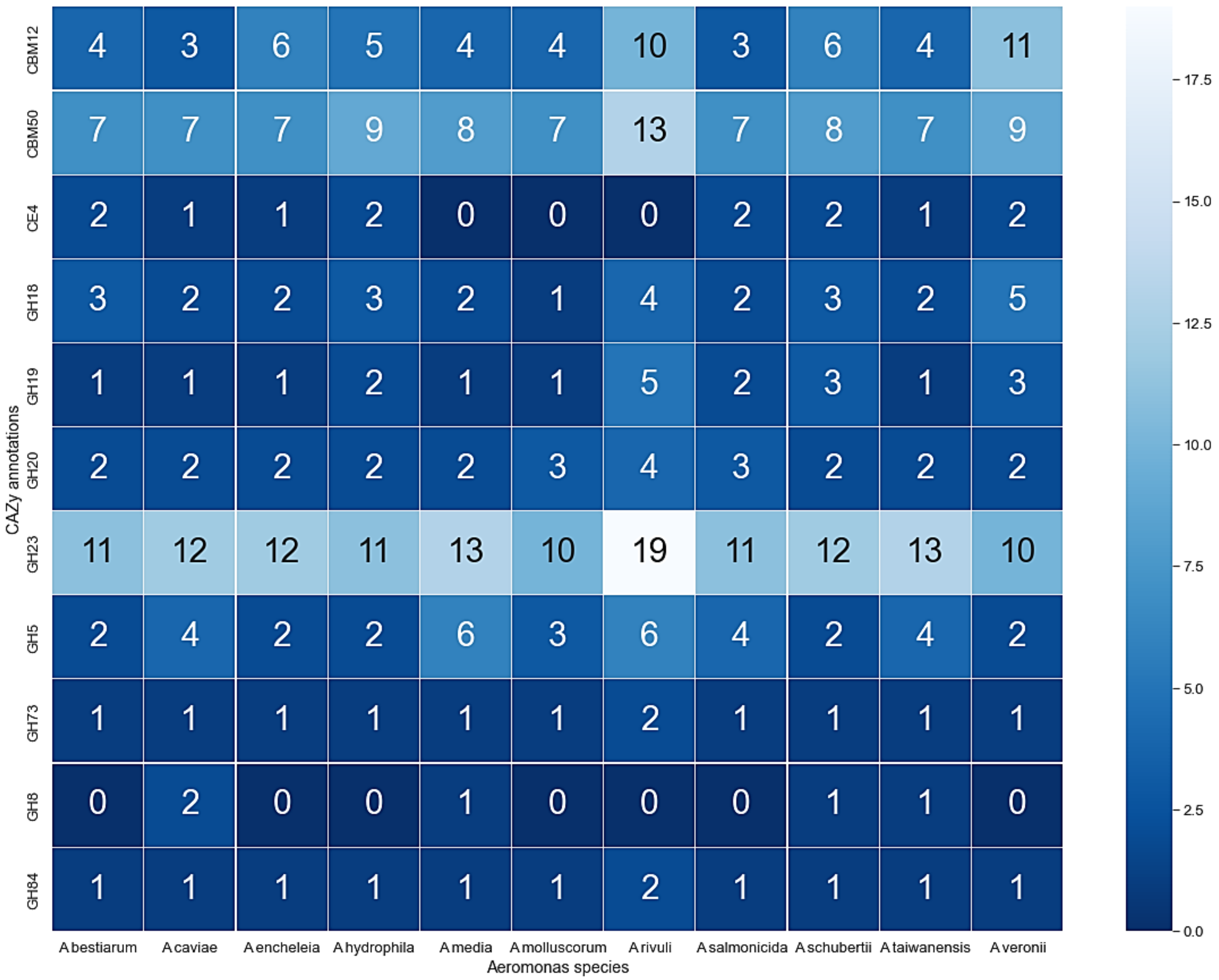

SI Figure 7: The heatmap shows the number of identified glycoside hydrolases (GH), related carbohydrate-binding modules (CBM) and chitin esterases (CE) in the genomes of different Aeromonas species. These are potentially involved in the breakdown of chitin and chitosan. The analyzed species are frequently found in drinking water distribution systems.

SI Table 3: The table lists CAZy database families used to search for the potential to degrade various carbohydrate biopolymers in Aeromonas genomes.

**Biopolymer**	**CAZy targets**
Chitin	CBM12, CBM14, CBM18, CBM50, CBM55, GH18, GH19, GH23, GH48, CBM32, GH18, GH20, GH73, GH84, GH85, GH89, GH111, GH116, GH163, GH5, GH7, GH8, GH46, GH75, GH80, CE4
Xylan	AA10, AA14, CBM2, CBM4, CBM6, CBM9, CBM13, CBM15, CBM22, CBM31, CBM35, CBM36, CBM42, CBM54, CBM59, CBM60, CBM72, CBM91, CE1, CE2, CE3, CE4, CE5, CE6, CE7, CE12, GH3, GH5, GH8, GH10, GH11, GH18, GH26, GH30, GH43, GH51, GH67, GH98, GH115, GH141, GT8, GT43, GT47, GT61
Cellulose	AA9, AA10, AA15, AA16, CBM1, CBM2, CBM3, CBM4, CBM6, CBM8, CBM9, CBM10, CBM16, CBM17, CBM28, CBM30, CBM37, CBM44, CBM46, CBM49, CBM59, CBM63, CBM64, CBM72, GH5, GH8, GT2
Starch	AA13, CBM20, CBM21, CBM25, CBM26, CBM34, CBM45, CBM53, CBM69, CBM74, CBM82, CBM83, GT5, GT35, GH13, GH14, GH57, GH126, GH15, GH57, GH97, GH119
Chitosan	GH3, GH5, GH7, GH8, GH18, GH46, GH75, GH80
